# Small-molecule control of neurotransmitter sulfonation

**DOI:** 10.1074/jbc.RA120.015177

**Published:** 2020-11-24

**Authors:** Ian Cook, Mary Cacace, Ting Wang, Kristie Darrah, Alexander Deiters, Thomas S. Leyh

**Affiliations:** 1 Department of Microbiology and Immunology, Albert Einstein College of Medicine, Bronx, New York, USA; 2 Department of Chemistry, University of Pittsburgh, Pittsburgh, Pennsylvania, USA

**Keywords:** catecholamine, neurotransmitter, sulfotransferase, allosteric, inhibitor, SULT1A3, molecular dynamics, human mammary epithelial cells, structure activity relationship, DOPAC, 3,4-dihydroxyphenylacetic acid, DP, dopamine, DPS, dopamine sulfate, DTT, dithiothreitol, GST, glutathione sepharose, HME, human mammary epithelial, 1-HP, 1-hydroxypyrene, HVA, homovanillic acid, MD, molecular dynamics, MDD, major depressive disorder, MEM, Minimum Essential Media, 3-MT, 3-methoxytyramine, PAP, 3′-phosphoadenosine-5′-phosphate, PAPS, 3′-phosphoadenosine 5′-phosphosulfate, SULT, sulfotransferase, Tam, 4-hydroxy-tamoxifen

## Abstract

Controlling unmodified serotonin levels in brain synapses is a primary objective when treating major depressive disorder—a disease that afflicts ∼20% of the world’s population. Roughly 60% of patients respond poorly to first-line treatments and thus new therapeutic strategies are sought. To this end, we have constructed isoform-specific inhibitors of the human cytosolic sulfotransferase 1A3 (SULT1A3)—the isoform responsible for sulfonating ∼80% of the serotonin in the extracellular brain fluid. The inhibitor design includes a core ring structure, which anchors the inhibitor into a SULT1A3-specific binding pocket located outside the active site, and a side chain crafted to act as a latch to inhibit turnover by fastening down the SULT1A3 active-site cap. The inhibitors are allosteric, they bind with nanomolar affinity and are highly specific for the 1A3 isoform. The cap-stabilizing effects of the latch can be accurately calculated and are predicted to extend throughout the cap and into the surrounding protein. A free-energy correlation demonstrates that the percent inhibition at saturating inhibitor varies linearly with cap stabilization — the correlation is linear because the rate-limiting step of the catalytic cycle, nucleotide release, scales linearly with the fraction of enzyme in the cap-open form. Inhibitor efficacy in cultured cells was studied using a human mammary epithelial cell line that expresses SULT1A3 at levels comparable with those found in neurons. The inhibitors perform similarly in *ex vivo* and *in vitro* studies; consequently, SULT1A3 turnover can now be potently suppressed in an isoform-specific manner in human cells.

Approximately one in five individuals worldwide will at some point suffer from major depressive disorder (MDD)—a depressive episode lasting two or more weeks (1). During such episodes, individuals are at 20-fold enhanced risk of suicide—the 10th leading cause of death in the United States (2). The penetrance of MDD is such that the World Health Organization predicts it will become the second leading cause of disease burden (second only to HIV) among the global populace by 2030 (3). Roughly one-third of patients with MDD achieve remission in response to treatment with a single first-line therapeutic, and the response is slow—remission requires approximately 7 weeks of treatment (4, 5). The suboptimal efficacy among first-line therapeutics has spurred extensive efforts in academia and industry to identify new and potentially more effective therapeutic strategies (4, 5, 6). In an effort to contribute to this issue, we have designed and synthesized compounds intended to control the sulfonation of catecholamine neurotransmitters in humans and to do so without significantly influencing the remainder of sulfonation metabolism. The design, synthesis, and testing of these inhibitors are described herein.

First-line MDD therapeutics are mainly used to increase synaptic levels of unmodified (i.e., active) serotonin (7). In microdialysates from human brain, serotonin is nearly completely oxidized by monoamine oxidase, and 80% of the oxidized metabolite is sulfonated (8); hence, monoamine oxidase and sulfotransferase (SULT) inhibitors are expected to act synergistically to increase the levels of unmodified serotonin.

Sulfotransferase 1A3 (SULT1A3) is responsible for the majority of neurotransmitter sulfonation in the central nervous system (9, 10). It is one of thirteen human SULT isoforms, each of which has had its catalytic efficiency (*k*_cat_/K_m_) “tuned” by evolution (10) toward a different area of metabolism (11). SULTs catalyze transfer of the sulfuryl (-SO_3_^-^) moiety from 3’-phosphoadenosine 5’-phosphosulfate (PAPS) to the hydroxyls and amines of hundreds, perhaps thousands of metabolites including scores of signaling small molecules. Sulfonation typically prevents signaling molecules from binding to their receptors and accelerates their clearance by enhancing their efficiency toward organic anion transporters (12, 13, 14).

In previous work (15), we discovered CMP8 (Fig. 1), which binds tightly (K_i_ = 34 nM) to SULT1A3 and allosterically inhibits its turnover. The structure of the ligand-bound enzyme (16) revealed that the inhibitor binds at a site that is unique to the 1A3 isoform. The site is situated outside the active site near the so-called catalytic cap of the enzyme (17, 18, 19), which must open and close during the catalytic cycle (20). At saturation, CMP8 reduces SULT1A3 turnover by a factor of 2. Here, using CMP8 as a template, we develop high-affinity, allosteric inhibitors that are specific for SULT1A3 and can virtually completely inhibit the isoform in cultured human cells.

**Figure 1:**
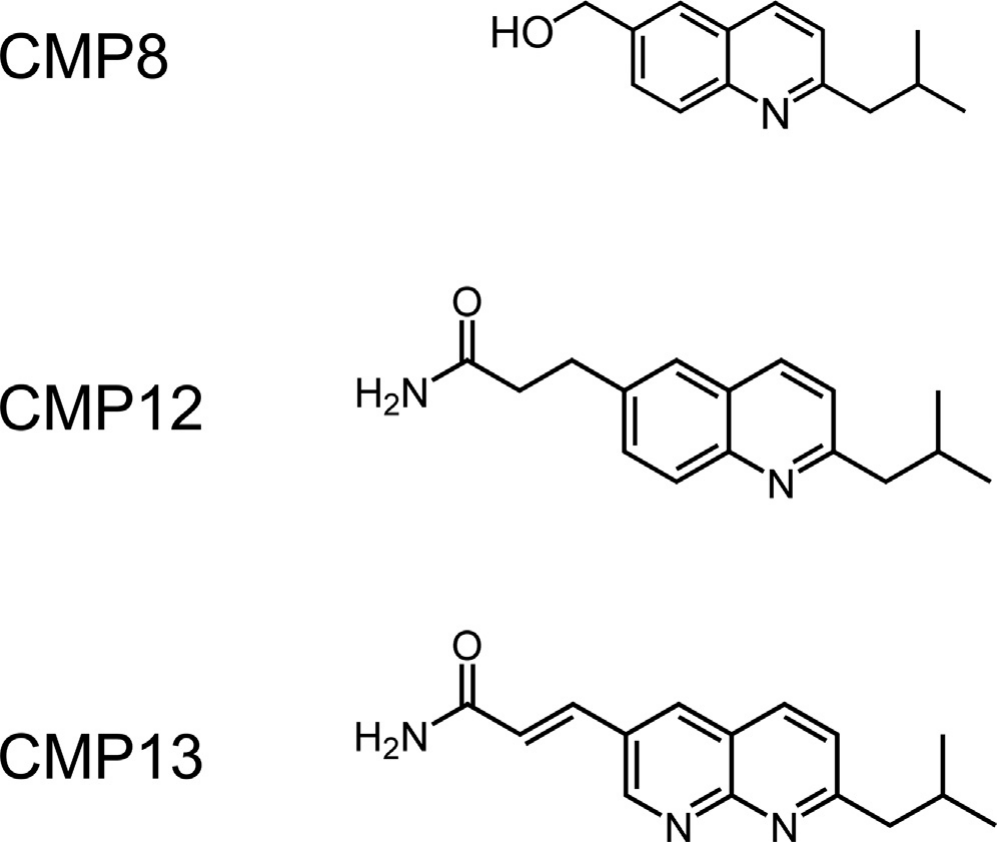
**The allosteric inhibitors.** CMP8, a reference compound in this study, has been described previously (15). The synthesis and characterization of CMP12 and CMP13 are described in Supporting Information.

## Results and discussion

### Inhibitor design

#### The active-site cap

SULTs harbor a conserved ∼30-residue active-site cap that encapsulates both the nucleotide donor (PAPS) and acceptor during the catalytic cycle. Molecular dynamics (MD) studies of cap closure (18, 19, 21) predict that a salt bridge forms between the 3′-phosphate of PAPS and a universally conserved cap residue (Lys249) early in the binding-and-closure reaction. The bridge remains intact as the nucleotide binds and appears to help draw the cap closed and fasten it shut as the binding reaction reaches completion. Once closed, nucleotide cannot escape the active site until the cap reopens (22); hence, the rate at which the nucleotide escapes the pocket is a linear function of the fraction of the time cap spends in the open position.

#### Design theory

The release of 3′-phosphoadenosine-5′-phosphate (PAP) from the cap-open conformation is the rate-limiting step in the forward reaction catalytic cycles of all SULTs studied to date (15, 22), including SULT1A3 (15); consequently, *k*_cat_ is expected to depend linearly on the fraction of the cap in the open position, *F*_*open*_, and it is given by the following equation:

(1) $$k_{cat}=F_{open}\;\cdot \;k_{rel}=\frac{E_{0}}{E_{0}\;+\;E_{C}}\;\cdot \;k_{rel}\;,$$

where *E*_*0*_ and *E*_*C*_ represent the cap-open and cap-closed forms of the PAP-bound enzyme, and *k*_*rel*_ represents the rate constant (15, 18) for nucleotide release from the cap-open form. *F*_*open*_ can be cast in terms of the cap-isomerization equilibrium constant, K_iso_ = [E_c_]/[E_o_], to yield the following:

(2) $$k_{cat}=\frac{1}{1\;+\;K_{iso}}\;\cdot \;k_{rel}$$

At K_iso_ >> 1

(3) $$k_{cat}\;∼\;\frac{k_{rel}}{K_{iso}}$$

Equilibrium-binding studies in the absence of inhibitors reveal that K_iso SULT1A3_ = 13 ± 1.7 (15, 18, 19). Inhibitors that stabilize the closed form of the cap (15, 23) will increase K_iso_; hence, the Equation 3 approximation is expected to hold for both inhibited and uninhibited enzyme forms.

In the case of SULT1A1 and SULT2A1, k_rel_ is independent of the binding of acceptors and cap-stabilizing allosteres; hence, opening the cap short-circuits communication between the allostere and nucleotide, which enters and exits the cap-open, allostere-bound enzyme with rate constants indistinguishable from those associated with the unliganded cap-open enzyme. If the SULT1A3 k_rel_ is also inhibitor independent, Equation 3 leads to the following *k*_cat_ ratio for any two inhibitors:

(4) $$\frac{k_{cat\;1}}{k_{cat\;2}}\;∼\;\frac{K_{iso\;2}}{K_{iso\;1}}$$

Thus, the relative cap stabilization of any two inhibitors is given by the ratio of their isomerization equilibrium constants, which, when couched in term of Gibbs potentials, yields the following equation:

(5) ΔΔGiso2−1=ΔGiso2−ΔGiso1=−RTln(Kiso2Kiso1)

Incorporating the Equation 4 approximation yields the following equation:

(6) ΔΔGiso2−1∼−RTln(kcat1kcat2)

which leads to the conclusion that differences in ground-state cap stability can be calculated from *k*_cat_ using the Eyring equation (24). Equation 6 predicts that ΔΔG_iso_ will be linear with changes in the net transition-state energetics calculated from *k*_cat_ and that the slope of such a correlation will equal one.

Our prior work with CMP8 (Fig. 1) revealed that when the C6-hydroxymethyl of the substituted quinoline is properly positioned in the binding site, which requires appropriate aliphatic spacing between the isobutyl moiety and the C2 ring position (15), the hydroxyl forms a hydrogen bond with a cap residue (Gln222) that stabilizes the closed form of the cap and inhibits turnover. At saturation, CMP8 suppresses turnover to 46% of that seen in its absence (15). Given that amide–amide interactions are predicted to be stable (25), we reasoned that replacing the hydroxyl group with an amido group might further inhibit turnover by strengthening bonding between the inhibitor and Gln222. A variety of C6-amide derivatives of CMP8 were considered, and two (CMP12 and CMP13, Fig. 1) seemed particularly promising on the basis of their predicted cap interactions, synthetic tractability and MD-predicted affinities; consequently, their effects on the energetics of the closed SULT1A3 cap were evaluated further.

**Figure 2:**
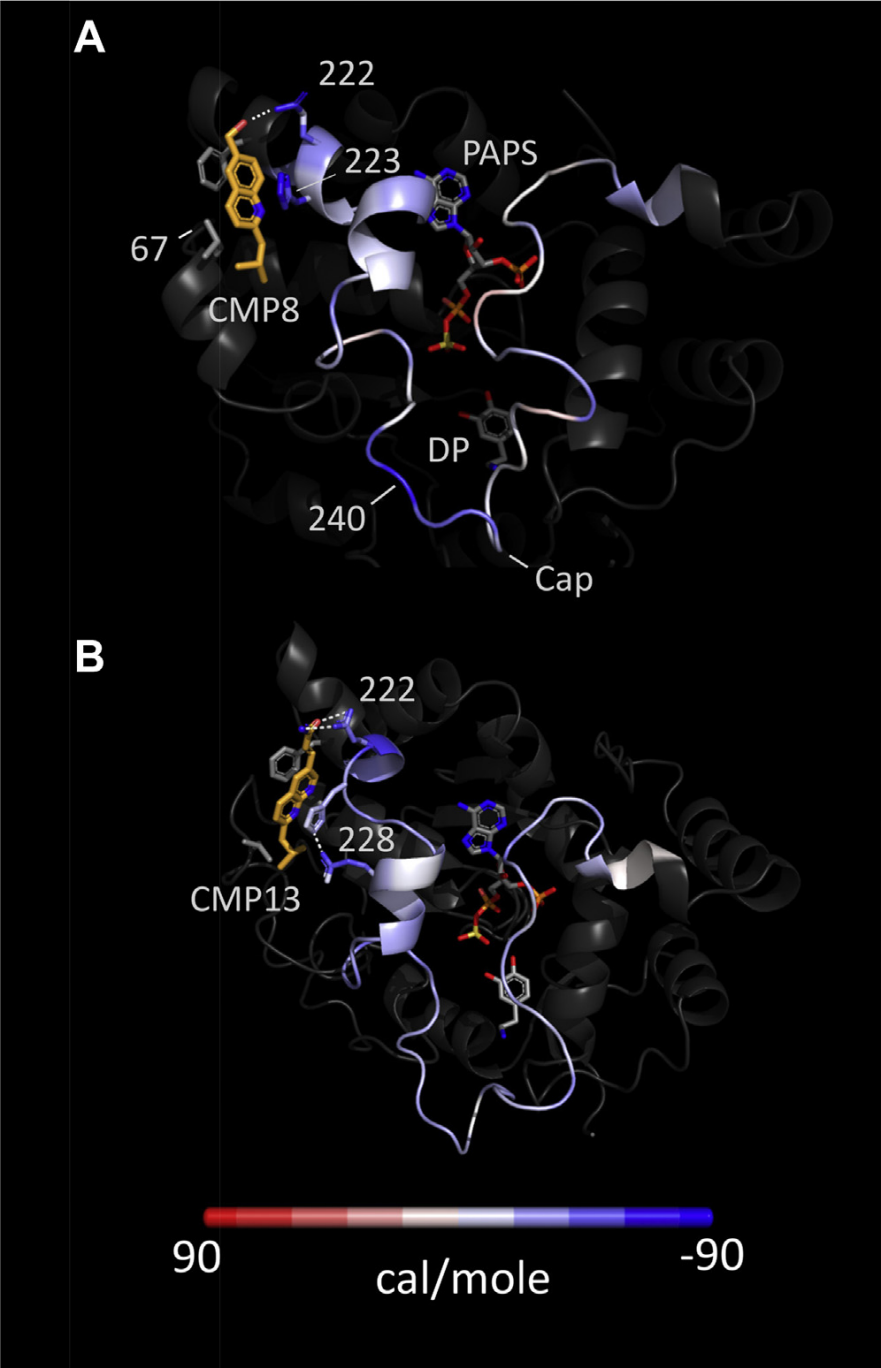
**Energy difference maps***. A,* the CMP8 map. The stabilizing effects of CMP8 are shown color-coded on the closed active-site cap of SULT1A3. Colors represent the changes in Gibbs potential that occur when CMP8 adds to the enzyme and correspond to the scale shown at the bottom of the figure. Numbers associated with the protein correspond to residue numbers. *B,* the CMP13 map. The Gibbs potential changes associated with replacing CMP8 with CMP13. SULT1A3, sulfotransferase 1A3.

The influence of CMP12 and CMP13 on cap energetics was calculated using *g_energy*—a GROMACS subroutine that defines the Gibbs potential of a given atom as the sum of the interaction energies between that atom and all other atoms (including solvent) within a 10-Å radius. The effects of ligand were calculated by comparing the energetics of two structures, a primary structure (which contains the ligand) and a reference structure, whose energetics are subtracted from those of the primary structure. Figure 2 presents the energy differences color-coded and “painted” onto the MD-predicted SULT1A3 cap. The effects of CMP8 are shown in Fig. 2*A*, for which the primary-structure ligands were dopamine (DP), PAPS, and CMP8; the reference lacked CMP8. As is evident, the energetic perturbations distribute throughout the cap, which is stabilized and destabilized in a regiospecific pattern. Notably, CMP8 effects are observed throughout the structure (not shown). Maximum stabilization occurs at two points of direct contact with CMP8 (Q222 and H223) and, curiously, at a distal residue (V240) situated in a dynamic cap region involved in acceptor selection (19). The effects of CMP13 are given in panel B, which illustrates the changes that occur when CMP13 is substituted for CMP8 (i.e., the panel B reference structure is the structure seen in panel A). CMP13 causes substantial stabilization beyond that provided by CMP8. The enhanced stabilization occurs throughout the cap with maximum stabilization again occurring at a direct contact point (Q222). As anticipated, CMP13 establishes an amide–amide interaction with Q222, presumably *via* the polar forms of the amide predicted by the AMBER force field (26). CMP13 also crimps together the two small helical elements seen in the upper left corner of the cap and thus stabilizes E228 by enabling it to hydrogen bond to H223.

The Gibbs free energy associated with all closed-cap atoms can be calculated by subtracting from the sum of the potentials of all atoms in the system (including water and ions) the potentials of all non-cap atoms. For CMP12 and CMP13, such calculations predict ΔG values of −1.17 and −1.41 kcal mol, respectively, which correspond to K_iso_ values of 7.6 and 11, and, in turn, 88% and 92% inhibition at saturating inhibitor—values that are substantially greater than the 54% obtained with CMP8. Given these predictions, CMP12 and CMP13 were synthesized (see Supplementary Information) and experimentally characterized.

#### *In vitro* characterization

**Figure 3:**
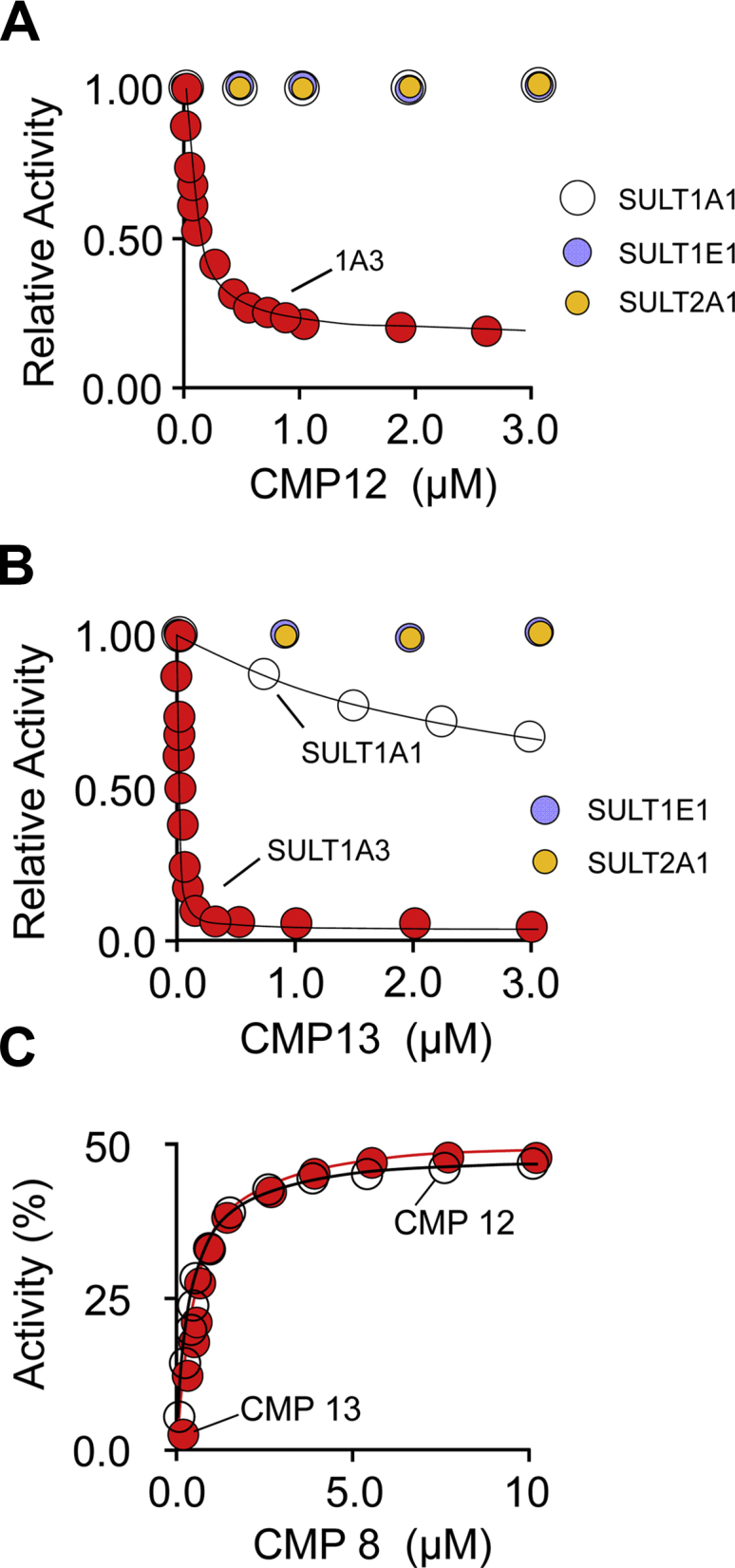
**Affinity, specificity, and binding sites of CMP12 and CMP13***. A* and *B,* inhibition studies. Initial rates of SULT-catalyzed 1-HP sulfonation are plotted as a function of inhibitor concentration. Rates are normalized to the rate observed in the absence of the inhibitor. Inhibition of the major SULT isoforms found in the brain and liver were tested. SULT activity was monitored *via* the sulfonation-dependent change in 1-HP fluorescence (λ_ex_ = 325 nm, λ_em_ = 375 nm (15, 28)). Reaction conditions were as follows: SULT (20 nM, active sites), PAPS (0.50 mM, 17 × K_m_), 1-HP (5.0 μM, 61 × K_m_), KPO_4_ (50 mM), pH 7.5, 25(±2) °C. Less than 5% of the concentration-limiting substrate was converted to the product at reaction endpoints. Each data point is the average of three independent determinations. The lines passing through the data are least-squares fits to a noncompetitive partial-inhibition model (see Experiment and Initial rate). *C,* CMP8 competes with CMP12 and CMP13*.* Initial rates at saturating CMP12 or CMP13 are plotted as a function of CMP8 concentration. Rates are normalized to the rate observed in the absence of the inhibitor. Rate measurements were performed as described for panels *A* and *B*. Reaction conditions were as follows: SULT1A3 (20 nM, active sites), CMP12 (1.4 μM, 20 × K_i_) or CMP13 (0.18 μM, 20 × K_i_), CMP8 (0–10 μM, 0–300 × K_i_), PAPS (0.50 mM, 17 × K_m_), 1-HP (5.0 μM, 61 × K_m_), KPO_4_ (50 mM), pH 7.5, 25(±2) °C. Lines passing through the data are the predictions of a noncompetitive partial-inhibition model in which inhibitors compete for the same site (see Experiment and Binding competition). SULT, sulfotransferase; PAP, 3′-phosphoadenosine-5′-phosphate; SULT1A3, sulfotransferase 1A3; PAPS, 3’-phosphoadenosine 5’-phosphosulfate; 1-HP, 1-hydroxypyrene.

The affinity and specificity of CMP12 and CMP13 were evaluated in initial-rate studies using the four major SULT isoforms expressed in the brain and liver (27)—SULT1A1, SULT1E1, SULT1A3, and SULT2A1. Initial-rate vs [inhibitor] plots are presented in Fig. 3, *A*–*B*. The data are normalized to the rates in the absence of inhibitor. Solid lines passing through the data are the predictions of least-squares fitting to a single-binding-site, partial-inhibition model. The best-fit parameters are found in Table 1. As is evident, both compounds bind tightly (K_i_ = 70 and 11 nM, respectively) and exhibit a high degree of specificity. CMP12 gave no detectable inhibition of SULTs 1A1, 1E1, and 2A1 at concentrations as high as 10 μM. CMP13 behaved similarly toward 1E1 and 2A1 and weakly inhibited 1A1 (K_i_ = 3.1 ± 0.2 μM). Both compounds are partial inhibitors, and their percent inhibition values at saturation (83 and 98%, respectively) agree well with the predicted values.

**Table 1:** *In vitro* and *ex vivo* inhibition parameters

CMP	*In vitro*		*Ex vivo*	
	K_i_ (nM)	% Inhibition at saturation	IC_50_ (nM)	% Inhibition at saturation
8^a^	34 (3.0)^b^	54 (3)		
12	70 (4.1)	83 (4)	260 (10)	84 (3)
13	11 (0.7)	98 (2)	12 (0.7)	95 (2)

a Values cited previously (15).

b Values in parentheses indicate 1-SD unit.

To confirm that CMPs 8, CMP12, and CMP13 bind to the same site, competitive binding of CMP8 against CMP12 and CMP13 was demonstrated in an initial-rate study in which CMP8 is titrated against saturating (20 × K_i_) concentrations of the competing inhibitor. Given that the percent inhibition at saturation of CMP8 (54%) is substantially less than that of either CMP12 or CMP13 (83 and 98%, respectively), competition predicts an alleviation of inhibition as the CMP8 concentration increases that plateaus at 54%, which is precisely what is observed (see Fig. 3*C*).

#### The inhibition mechanism

To assess whether PAP release from cap-open forms of SULT1A3 is independent of the inhibitor, and thus conforms to the design theory outlined previously, PAP/CMP13 interactions were evaluated using equilibrium and pre–steady-state binding studies. In certain studies, 4-hydroxy-tamoxifen (Tam) was used to sterically “hold” the cap open. Tam binds at the acceptor pocket and is too large to bind to the cap-closed enzyme; hence, at saturation, it “fastens” the cap open, which allows cap-open binding studies with ligands that would otherwise close it. In effect, Tam “stalls” the binding reaction at a point where ligand has bound but the cap has not yet closed. It should be noted that the affinities of PAP for E (the unliganded open form) and E·TAM are indistinguishable (15, 28); hence, these ligands only interact indirectly through their effects on the open/closed status of the cap.

**Figure 4:**
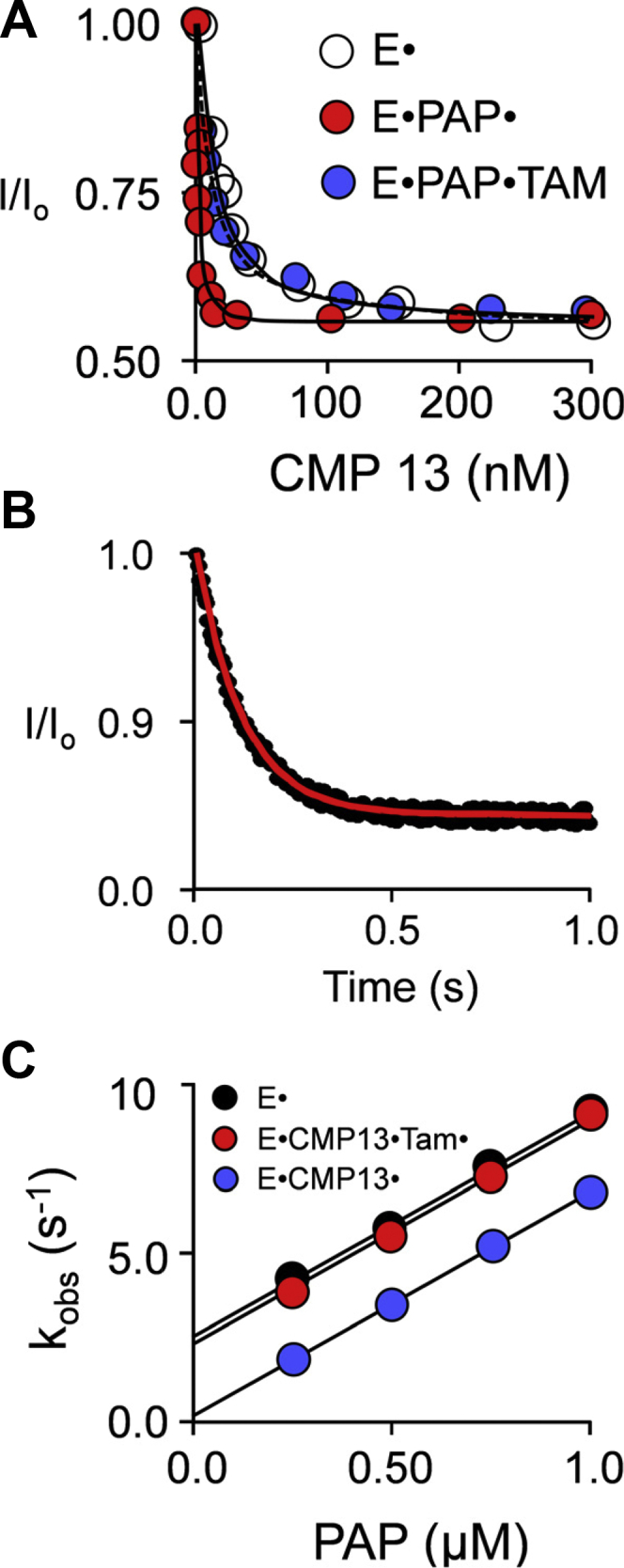
**The inhibition mechanism***. A,* equilibrium binding. Binding was monitored *via* ligand-induced changes in SULT1A3 intrinsic fluorescence (λ_ex_ = 290 nm, λ_em_ = 335 nm). CMP13 was titrated into a solution containing SULT1A3 (15 nM, dimer), PAP [0 μM (*white dots*) or 350 μM, 100 × K_d_ (*red and blue dots*)], Tam [0 μM (*black and red dots*) or 160 μM, 200 × K_d_ (*blue dots*)], KPO_4_ (50 mM), pH 7.5, 25(±2) °C. Each dot represents the average of three independent titrations. Lines passing through the data represent the outcomes predicted by least-squares fitting of the averaged data to a single-binding-site model. *B,* representative PAP-binding reaction. PAP binding was monitored using a stopped-flow fluorimeter (λ_ex_ = 290 nm, λ_em_ ≥ 330 nm (cutoff filter)). Reactions were initiated by rapidly mixing (1:1 v/v) a solution containing SULT1A3 (30 nM, dimer), CMP13 (1.0 μM, 100 × K_d_), Tam (160 μM, 200 × K_d_), KPO_4_ (50 mM), pH 7.5, 25(±2) °C, with a solution that was identical except that it lacked SULT1A3 and contained PAP (2.0 μM). The average of five independent progress curves shown and the *k*_*obs*_ was obtained by fitting the data to a single exponential. *C,* pre–steady-state binding*.* PAP binding was monitored as described for *panel B*. Reactions were initiated by mixing (1:1 v/v) a solution containing SULT1A3 (30 nM, dimer), CMP13 [0 μM (*black dots*) or 1.0 μM, 100 × K_d_ (*red and blue dots*)], Tam [0 μM (*black and blue dots*) or 160 μM, 200 × K_d_ (*red dots*)], KPO_4_ (50 mM), pH 7.5, 25(±2) °C, with a solution that was identical except that it lacked SULT1A3 and contained PAP at twice the indicated concentrations. Each *k*_*obs*_ value was determined in triplicate, and the averaged values are shown. *k*_*on*_ and *k*_*off*_ are given, respectively, by the slopes and intercepts obtained from linear least-squares fitting of the *k*_*obs*_ vs [PAP] plot. PAP, 3′-phosphoadenosine-5′-phosphate; SULT1A3, sulfotransferase 1A3; Tam, 4-hydroxy-tamoxifen.

The binding of CMP13 to E, E·PAP, and E·PAP·Tam is shown in Fig. 4*A*. PAP and Tam are saturating (100 and 200 × K_d_, respectively) in these studies. The lines passing through data are the result of least-squares fitting to a single-site binding model, and the best-fit binding parameters are found in Table 2. Consistent with cap-stabilizing interactions, CMP13 binds the cap-closed enzyme (E·PAP, K_d_ = 11 nM) 12-fold more tightly than the open form (E, K_d_ = 130 ± 8 nM). To address the question of whether CMP13 and PAP interact in the cap-open form, the affinity of CMP13 for the E·Tam·PAP complex was compared with that for the unliganded cap-open form (E)—the affinities are indistinguishable (130 ± 8 nM and 133 ± 8 nM, respectively); hence, the ligands do not communicate in the cap-open complexes.

**Table 2:** CMP13 binding to SULT1A3 complexes

	Complex		
	E	E·PAP	E·PAP·Tam
K_d_ (nM)	130 (8)^a^	11 (0.4)	133 (8)

PAP, 3′-phosphoadenosine-5′-phosphate; SULT1A3, sulfotransferase 1A3; Tam, 4-hydroxy-tamoxifen.

a Values in parentheses indicate 1-SD unit.

Although the foregoing findings reveal that CMP13 and PAP do not interact in cap-open complexes, they do not address the possibility that the inhibitor causes the PAP on- and off-rate constants to vary in fixed ratio, which will occur if CMP13 stabilizes the cap to the same extent in the presence and absence of nucleotide. To resolve this uncertainty, PAP on- and off-rate constants for a series of open and closed complexes were determined using stopped-flow fluorescence. A representative binding reaction can be seen in Fig. 4*B*. The k_obs_-*vs*-[PAP] plots obtained from the studies are given in Fig. 4*C*. The binding reactions were pseudo-first order in [PAP], and k_obs_ values were obtained by fitting reaction progress curves to a single-exponential equation. On- and off-rate constants (see Table 3) are given by the plot slopes and intercepts.

**Table 3:** PAP binding to SULT1A3 complexes

Complex	k_on_ (μM^-1^s^-1^)	k_off_ (s^-1^)	K_d_^a^ (μM)	K_d_^b^ (μM)
E	6.9 (0.3)^c^	2.5 (0.3)	0.36	0.32 (0.04)
E·CMP13	6.6 (0.2)	0.18 (0.07)	0.029	0.031 (0.002)
E·CMP13·Tam	6.5 (0.3)	2.3 (0.2)	0.35	0.35 (0.03)

PAP, 3′-phosphoadenosine-5′-phosphate; SULT1A3, sulfotransferase 1A3; Tam, 4-hydroxy-tamoxifen.

a K_d_ was determined using the k_off_/k_on_.

b K_d_ was determined by equilibrium binding.

c Values in parentheses indicate 1-SD unit.

The pre–steady-state data reveal that PAP adds to both the cap-open (E, black dots) and inhibitor-bound (E·CMP13, blue dots) enzyme with virtually identical on-rate constants (Table 3); thus, the cap is largely open in the inhibitor-bound forms. The effect of an inhibitor is seen only on the nucleotide off-rate constant (i.e. intercepts) which decreases ∼14-fold—a value consistent with the PAP–CMP13 interactions seen in the equilibrium-binding studies. Adding Tam (E·CMP13·Tam, red dots), which “lifts” the cap, causes the PAP release rate constant to become identical to that for the open form (E). Given these findings, we conclude that inhibitor alone does not detectably close the cap and its binding does not influence the off-rate constant of the nucleotide from the cap-open form.

**Figure 5:**
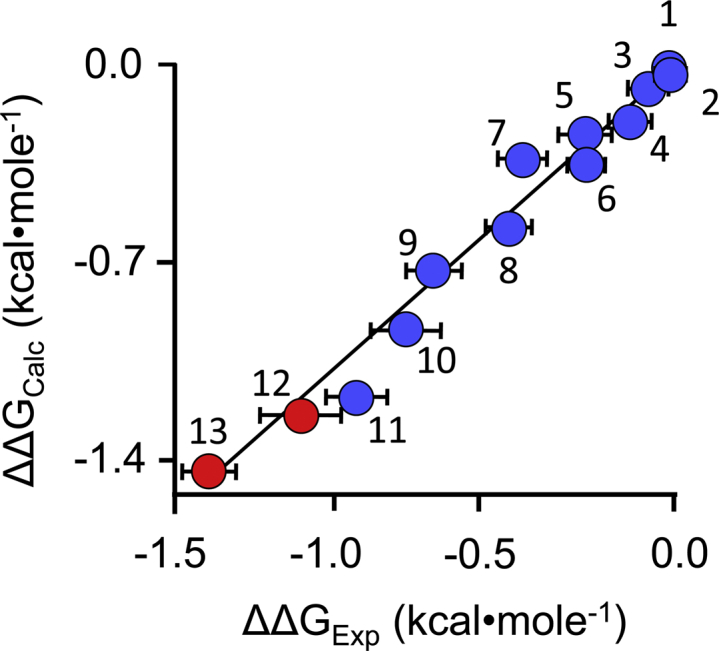
**Free-energy correlation**. Calculated and experimentally determined ΔΔG values for inhibitor-induced stabilization of the SULT1A3 cap are plotted vs one another. The closed-cap form of the SULT1A3·DP·PAPS complex, which lacks an inhibitor, was used as the reference structure in the MD calculations. The datasets correlate linearly with slope = 1.1 (R = 0.99). *Red dots* indicate the new compounds described herein; *blue dots* identify compounds described in a previous study (15). The numbering corresponds to the structures seen in Table 1 and the previous study. Errors in the calculated (Y-axis) dimension are minute. SULT1A3, sulfotransferase 1A3; PAPS, 3’-phosphoadenosine 5’-phosphosulfate; DP, dopamine; MD, molecular dynamics.

The pre–steady-state findings confirm that the rate constant governing PAP release from the cap-open enzyme is not affected by the inhibitor, and thus, the inhibition mechanism conforms to the design theory. Figure 5 correlates the predicted changes in cap-stabilization free energy across a series of inhibitors (ΔΔG_Calc_) against their experimentally determined changes in transition-state free energies (ΔΔG_Exp_) calculated using *k*_cat_ (see Design theory). The changes in transition-state energetics reflect changes in the cap isomerization equilibrium constants (see Equations 6 and 7). The free-energy correlation combines the results from the current studies (red dots) with those from a previous study of CMP8-related inhibitors (blue dots) (15). The correlation is linear with a slope of 1.1 ± 0.1, which indicates not only that changes in the ligand-induced cap stabilization can be calculated reliably but also that the full complement of cap-stabilizing chemical potential is coupled to inhibition.

#### *Ex vivo* characterization

A stable transfectant cell line was constructed to test the ability of CMP12 and CMP13 to suppress catecholamine sulfonation in cultured human cells. Human mammary epithelial (HME) cells were selected because these cells are DP responsive (29), they do not express SULT1A3 at levels detected by Western blotting (30), and HME cell extracts do not detectibly sulfonate DP (30). HME cells were transfected with plasmid containing the coding region of either SULT1A3 or a negative control (14) (see Experiment and Transfection protocol). Transfectant isolates were grown to near confluence and harvested, and cell extracts were prepared (see Experiment). SULT1A3 levels in the extracts were assessed by determining SULT activity *per* μg extract (see Experiment). Activity in extracts of the SULT1A3-positive transfectants ranged from 1 to 85 times the levels present in negative-control extracts. SULT1A3 levels in extracts of SH-SY5Y cells (a human dopaminergic cell line (31)) and human platelets (where SULT1A3 levels are comparable to those in the central nervous system (32)) are roughly 40 times the levels in the HME negative-control extracts. Consequently, a transfectant, 1A3(+) with extract activity 42 times that of the negative-control isolate, 1A3(-), was selected for further experiments.

**Figure 6:**
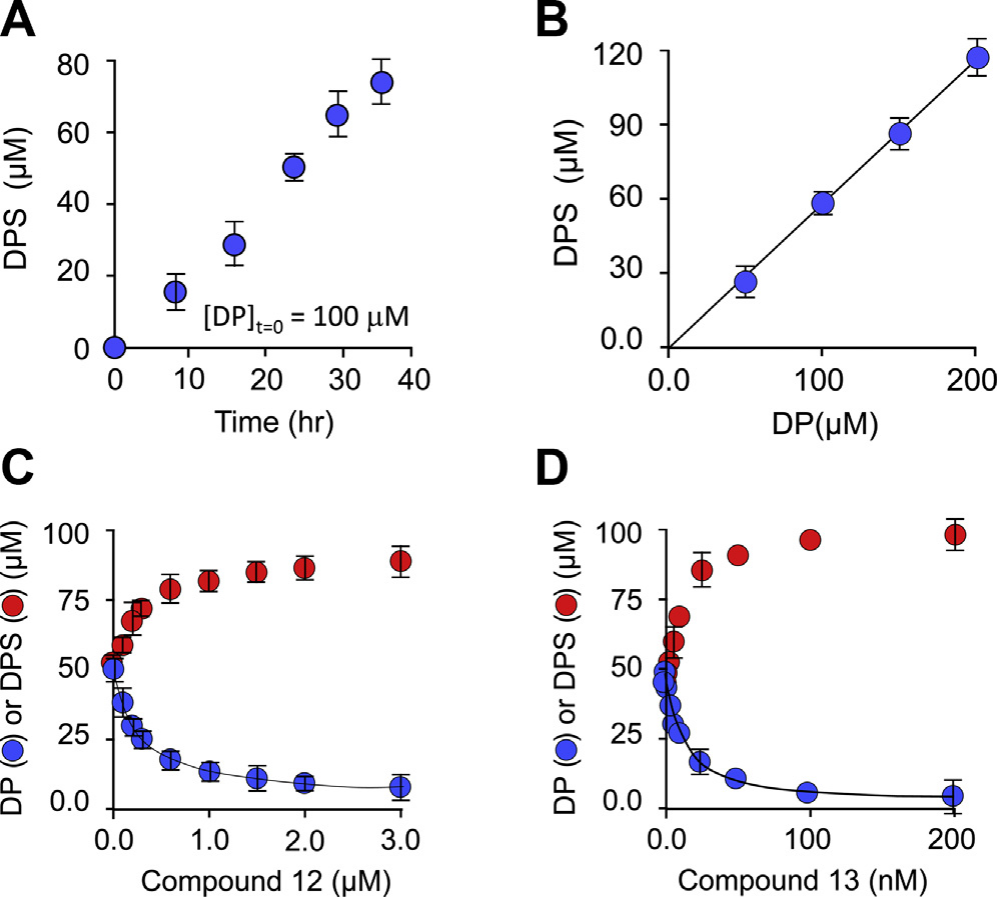
**HME(+)-cell DPS synthesis and inhibition**. *A,* Time dependence of DPS formation. DP was added at 100 μM to the growth medium of 60 to 70% confluent HME(+)-cells, and the DPS concentration in the medium was determined at the indicated time intervals. *B,* DP-concentration dependence of DPS synthesis. The Y-axis indicates the concentration of DPS in the growth media 24 h after DP addition. The X-axis indicates the DP concentration added at t = 0 to the growth medium of 60 to 70% confluent HME(+)-cells. *C* and *D,* Inhibition of DPS synthesis*.* DP was added at 100 μM to the HME(+)-cell growth media containing the inhibitor at the indicated concentrations. The levels of DP (*red dots*) and DPS (*blue dots*) were determined 24 h after DP addition. The solid line through the DP data is the outcome predicted by least-squares fitting using the following inhibition model: [DPS] = [DPS]_-inh_ – [[DPS]_sat’d inh_ × [I]/(IC_50_ + [I])]. *A–D*, dopamine metabolites were separated and quantitated using HPLC (see Experiment); each data point represents the average of three independent determinations, and the sum of DP and DPS concentrations was ≥95% of the total added DP. DP, dopamine; DPS, dopamine sulfate; HME, human mammary epithelial.

To establish conditions for *ex vivo* inhibition studies, the DP concentration and time dependence of dopamine sulfate (DPS) synthesis by 1A3(+) cells were assessed. DP and its metabolites (DPS, 3-methoxytyramine [3-MT], 3,4-dihydroxyphenylacetic acid [DOPAC], and homovanillic acid [HVA]) are baseline separable using HPLC and can be quantitated optically at 280 nM (see Experiment and Fig. S1). Only DP and DPS were detected in the cell media, and together, they constituted >95% of the DP initially added to the media. The DPS concentration in the 1A3(+) media at 24 h after DP addition is plotted as a function of DP concentration in Fig. 6*B*. As is evident, the dose-response curve is linear at concentrations as high as 200 μM DP. Panel B demonstrates that DPS production (at 100 μM DP) is linear with time out to 36 h; notably, DPS formation is linear with time at each of the four DP concentrations associated with panel A (Fig. S2). Linearity with time and DP concentration indicates that DP uptake, conversion of DP to DPS, and DPS export are in a steady state and subsaturated with DP; thus, at 100 μM DP, the system is expected to respond linearly (1:1) with SULT1A3 inhibition.

The results of the HME(+) cell inhibition studies can be seen in Fig. 6, *C*–*D*, and the inhibition parameters are compiled in Table 1. The lines through the DPS datasets (blue dots) are the outcomes predicted using IC_50_ value obtained by fitting the data using the following equation: [DPS] = [DPS]_- inh_ – ([DPS]_sat’d inh_ × [I]/(IC_50_ + [I]) (33). The compounds performed well in *ex vivo* studies—their IC_50_ and K_i_ values are comparable (Table 1) and their percent inhibition at saturation is identical, within error, to that obtained from the initial-rate studies. The nearly coincident percent-inhibition-at-saturation values suggest that SULT1A3 turnover rate limits DPS accumulation in the media, and, as has been found elsewhere (14, 34, 35), that export of the sulfated metabolite is fast relative to its formation. Thus, it appears that flux through the major neurotransmitter sulfonation pathway can be controlled in cell-based studies and perhaps also in animals.

## Conclusions

Isoform-specific SULT1A3 allosteric inhibitors have been designed, synthesized, and evaluated *in vitro* and *ex vivo*. The quinoline and naphthyridine cores of the inhibitors engender isoform specificity by anchoring the ligands into a SULT1A3-specific binding site situated outside of the active site; hence, the inhibitors are allosteric (i.e., noncompetitive) and their effects cannot be “washed out” by competition versus substrates that accumulate upstream of the point of inhibition. The inhibitor elements directly engaged in slowing turnover are the substituents that tether the cores to the active-site cap, which must open and close during the catalytic cycle. The tethers were designed to slow turnover by stabilizing the cap-closed conformation.

GROMACS proved remarkably accurate in predicting substituent effects on the energetics of cap closure. The predictions were born out in a free-energy correlation between calculated cap-stabilization free energy and turnover, and in mechanism studies, which showed that the substituents do indeed inhibit *via* cap stabilization.

*In vitro* and *ex vivo* studies reveal that the inhibitors are allosteric, that they inhibit with nanomolar affinities, and that they are highly specific for SULT1A3 over the other major SULT isoforms found in the liver and brain. The upshot of these studies is that it is now possible to potently and isoform specifically inhibit SULT1A3 in human cells.

## Materials

The materials and sources used in this study are as follows: dithiothreitol (DTT), EDTA, L-glutathione (reduced), 1-hydroxypyrene (1-HP), imidazole, IPTG, lysozyme, pepstatin A, and potassium phosphate were the highest grade available from Sigma. Ampicillin, DOPAC, DP, formic acid, HVA, HME cells, KCl, KOH, LB media, 3-MT, MgCl_2_, Minimum Essential Media (MEM), neomycin, tris(hydroxymethyl) amino-methane (Tris) base, Ultra PFPP column (150 × 4.6 mm length, 3 μm bead) (Restek Corp.), and PMSF were purchased from Fisher Scientific. Glutathione- and nickel-chelating resins were obtained from GE Healthcare. Lipofectamine and Opti-MEM were purchased from EMD Millipore Corporation. ^35^S-sulfate was purchased from PerkinElmer. Competent *Escherichia coli* (BL21(DE3)) was purchased from Novagen. Synthesis and purification of PAPS and PAP is previously described (36), and purity, determined by anion-exchange HPLC, was ≥99%.

### Computer and software

MD simulations were performed on a Parallel Quantum Solutions QS32-2670C-XS8 computer. PQS Molecular Builder was purchased from Parallel Quantum Solutions (37). The source code for GROningen MAchine for Chemical Simulation (GROMACS) 4.5 was downloaded from http://www.GROMACS.org under the GROMACS General Public License (GPL). *Antechamber* was acquired as part of AmberTools, under the GNU General Public License. A Genetically Optimized Ligand Docking license was obtained from the Cambridge Crystallographic Data Center.

## Experimental procedure

### SULT purification

*E. coli*–optimized SULT coding regions were inserted into a pGEX-6P expression vector that fuses a triple-tag (*N*-His/GST/MBP) PreScission protease-cleavable protein to the SULT *N*-terminus. *E. coli* (BL21(DE3)) harboring an SULT expression plasmid was grown at 37 °C in the LB medium (15, 28, 38). At OD_600_ ∼ 0.6, the culture was temperature shifted to 17 °C by swirling flasks in an ice/water bath. On reaching 17 °C, IPTG was added (0.30 mM) to the culture, and it was incubated at 17 °C for 18 h. Cells were then pelleted, resuspended in the lysis buffer (PMSF (290 μM), pepstatin A (1.5 μM), lysozyme (0.10 mg/ml), EDTA (2.0 mM), KCl (400 mM), K_2_PO_4_ (50 mM), pH 7.5), sonicated, and centrifuged (10,000g, 1.0 h, 4 °C). MgCl_2_ (5.0 mM) was added to chelate EDTA before passing the solution through a Chelating Sepharose Fast Flow column charged with Ni^2+^. The column was washed (imidazole (10 mM), KCl (400 mM), and KPO_4_ (50 mM), pH 7.5), and the enzyme was eluted (imidazole (250 mM), KCl (400 mM), and KPO_4_ (50 mM), pH 7.5) and loaded directly onto a glutathione sepharose (GST) column. The GST column was washed (DTT (2.0 mM), KCl (400 mM), and KPO_4_ (50 mM), pH 7.5) before eluting the tagged enzyme (reduced glutathione (10 mM), DTT (2.0 mM), KCl (400 mM), and Tris-Cl (100 mM), pH 8.0). The fusion protein was digested overnight at 4 °C using PreScission Protease and passed through a GST column to remove the tag. Cleavage leaves Gly-Pro at the SULT N-terminus. The proteins were ≥95% pure as judged by SDS-PAGE. The proteins were concentrated, and their concentrations were determined optically (Ɛ_280_ = 53.9, 51.5, 68.6, and 73.4 mM^-1^ cm^-1^ for 1A3, 1A1, 1E1, and 2A1, respectively) before being flash-frozen (dry ice/ethanol) and stored at −80 °C.

### Dynamic docking

MD docking of CMP12 and CMP13 was performed as described previously (15). Briefly, a ligand-free homology model of SULT1A3 was constructed from the SULT1A3·PAP·DP structure (PDB) code 2A3R (9) using SWISS-MODEL (39). The model was protonated at pH 7.4 and energy-minimized using GROMACS 4.5 (40) using Amber. Generalized AMBER force-field energy parameters for CMP12 and CMP13 were created using Antechamber (26) as described previously (15). The *improper*_*dihedral_restraint* function in GROMACS was used to maintain resonance between the CMP13, C6 R-group, and ring system. The function was parameterized to enforce an energetic penalty (200 kJ mol^-1^ rad^-1^) for nonplanarity. The ligands (PAPS, DP, and CMP12 or CMP13) were positioned initially using Genetically Optimized Ligand Docking (41, 42). The system was then equilibrated (NaCl (50 mM), pH 7.4, 298 °K) in 100-ps increments using GROMACS. Once the RMS deviation of the system is stabilized, indicating that equilibrium had been reached, equilibrium was confirmed by ensuring that the RMS remained stable over an additional 10 ns. The time-averaged equilibrated structure was used as the start point for all g_energy calculations, which were performed over a 10-ns time frame using a 2-ps step size (40).

### Initial rate

Initial-rate inhibition parameters were obtained from classical initial-rate studies (16, 28, 43). Reactions were initiated by addition of PAPS (0.50 mM, 17 × K_m_) to a solution containing SULT1A3 (20 nM, active sites), inhibitor (0.2–20 × K_i_), 1-HP (5.0 μM, 60 × K_m_), and KPO_4_ (50 mM), pH 7.5, 25(±2) °C. Reaction progress was monitored *via* the fluorescence change associated with 1-HP sulfonation (λ_ex_ = 325 nm, λ_em_ = 370 nm (15, 28)). Less than 5% of the 1-HP was converted to the product at reaction endpoints. K_i_ and *k*_cat inh_ (i.e., turnover at saturating inhibitor and substrate) were obtained by fitting to the following quadratic equation for noncompetitive, partial inhibition at [S] >> K_m_ (44):

(7) $${\text{v/V}}_{\text{max}}=([{\text{E}}_{\text{tot}}]+[\text{ESI}](\alpha -1))/[{\text{E}}_{\text{tot}}]$$

where

(8) $$\alpha =k_{\text{cat inh}}/k_{\text{cat}}$$

and

(9) $$[\text{ESI}]=[({\left[\text{E}\right]}_{\text{tot}}\;+\;{\left[\text{I}\right]}_{\text{tot}}\;+\;{\text{K}}_{\text{i}})\;-\;{{\left[({\left[\text{E}\right]}_{\text{tot}}\;+\;{\left[\text{I}\right]}_{\text{tot}}\;+\;{\text{K}}_{\text{i}}\right)}^{2}\;-\;4\cdot {\left[\text{I}\right]}_{\text{tot}}\cdot {\left[\text{E}\right]}_{\text{tot}}]}^{1/2}]/2$$

### Equilibrium binding

CMP13 binding to SULT1A3 was monitored *via* binding-induced changes in SULT1A3 intrinsic fluorescence (λ_ex_= 290 nm, λ_em_= 335 nm (17)). Typical conditions were as follows: SULT (15 nM, dimer), CMP13 (0.10–20 × K_d_), PAP (0 or 350 μM, 100 × K_d_), Tam (0 or 160 μM, 200 × K_d_), KPO_4_ (50 mM), pH 7.5, 25(±2) °C. PAP binding was determined using the same protocol except that [PAP] was varied and CMP13 was 1.0 μM (100 × K_i_). Titrations were performed in triplicate. Data were averaged and least-squares fit using a model that assumes a single binding site *per* monomer.

### Pre–steady-state binding

Pre–steady-state binding of PAP to SULT1A3 was monitored *via* ligand-induced changes in SULT1A3 fluorescence using an Applied Photophysics SX20 stopped-flow spectrofluorometer. Fluorescence was measured at λ_ex_ = 290 nm and λ_em_ ≥ 330 nm (using a cutoff filter). A solution containing SULT1A3 (20 nM, dimer), CMP13 (0 or 1.0 μM, 100 × K_i_), Tam (0 or 160 μM, 200 × K_d_), and KPO_4_ (50 mM), pH 7.5, was rapidly mixed (1:1, v:v) with a solution that was identical except that it contained PAP (0.5, 1.0, 1.5, or 2.0 μM) and did not contain enzyme. All reactions were pseudo first order with respect to [PAP]. k_obs_ values, determined in triplicate at each PAP concentration, were obtained by least-squares fitting the average of five progress curves to a single-exponential equation. k_on_ and k_off_, obtained by linear least-squares fitting, are given by the slopes and intercepts of four-point k_obs_-*vs*-[PAP] plots.

### Binding competition

Reactions were initiated by addition of PAPS (0.50 mM, 17 × K_m_) to a solution containing SULT1A3 (20 nM, active sites), CMP12 (1.4 μM, 20 × K_i_) or CMP13 (0.18 μM, 20 × K_i_), CMP 8 (0–10 μM, 0–300 × K_i_), 1-HP (5.0 μM, 60 × K_m_), and KPO_4_ (50 mM), pH 7.5, at 25(±2) °C. Reaction progress was monitored *via* the fluorescence change associated with 1-HP sulfonation (λ_ex_ = 325 nm, λ_em_ = 370 nm (15, 28)). Initial rates were performed in triplicate as described previously.

To confirm that the data conform to a partial-noncompetitive inhibition model, the experimental outcome was simulated and the simulation was compared with the experimental results. The simulation was performed using the following algebraic model, in which both substrates are saturating and the inhibitors (A and B) bind competitively at the allosteric site (1):

(10) $$\text{v}\;=\;{\text{V}}_{\text{max}}\cdot ({\text{K}}_{\text{iA}}\cdot {\text{K}}_{\text{iB}}\;+\;({\text{K}}_{\text{iB}}\cdot \alpha_{\text{A}})\cdot [\text{A}])\;+\;({\text{K}}_{\text{iA}}\cdot \alpha_{\text{B}})\cdot [\text{B}])/({\text{K}}_{\text{iA}}\cdot {\text{K}}_{\text{iB}}\;+\;[\text{A}]\cdot {\text{K}}_{\text{iB}}\;+\;[\text{B}]\cdot {\text{K}}_{\text{iA}})$$

The constants can be found in Table 1 and reference (15). α_A_ and α_B_ are the fraction of V_max_ at saturating inhibitor (A or B), and K_iA_ and K_iB_ are the inhibition constants. At a fixed concentration of the inhibitor (eg., A), Equation 7 simplifies to the following:

(11) $$\text{v}={\text{V}}_{\text{max}}\cdot ({\text{C}}_{1}\cdot {\text{K}}_{\text{iB}}\;+\;\alpha_{\text{B}}\cdot [\text{B}])/({\text{C}}_{2}\cdot {\text{K}}_{\text{iB}}\;+\;[\text{B}])$$

where

(12) $${\text{C}}_{1}=1\;+\;[([\text{A}]\cdot \alpha_{\text{A}})/{\text{K}}_{\text{iA}}]\;\text{and}\;{\text{C}}_{2}=\;1\;+\;([\text{A}]/{\text{K}}_{\text{iA}})$$

The results of the simulations can be seen overlain on the binding-competition data in Fig 3C.

### Transfection protocol

HME cells are grown to confluency at 37 °C in the MEM on 10-cm tissue culture plates. Cells are then washed (3×) with PBS media (25 °C) before being coated with a Lipofectamine-DNA solution: Lipofectamine (2.5 units/ml), Opti-MEM, pcDNA 3.1 harboring an SULT1A3 or negative control (14) coding region (50 μg/ml), 37 °C. Twenty-four hours later, cells are washed (3x) with PBS (25 °C) before adding the MEM containing neomycin (400 μg ml^−1^) to select stable transfectants. Cells are grown under selective pressure at 37 °C until single colonies are visible (MEM/neomycin is replenished every 48 h). Single colonies are transferred to 12-well plates and grown at 37 °C for further experimentation and storage.

### SULT1A3 levels in transfectant extracts

Transfectants are grown at 37 °C to 60 to 70% confluency in 12-well plates, washed (3×) with PBS (25 °C), and lysed using RIPA buffer (0.50 ml) (14). The lysate is centrifuged (15k *g*, 10 min, 25 °C), and the supernatant is removed, flash-frozen (dry ice/ethanol), and stored as extract at −80 °C. Extract protein concentrations are determined using the Bradford assay (45), and SULT levels are determined by measuring turnover at saturating substrate concentration. Assay conditions are identical to those described in *Initial rate* except that extract (1–3 μg) is added in lieu of pure enzyme.

### DPS extinction coefficient

To our knowledge, the extinction coefficient of DPS has not been reported. To determine the coefficient, [^35^S]-DPS was enzymatically synthesized using [^35^S]-PAPS that was synthesized enzymatically using [^35^S]-SO_4_ (20, 36). The [^35^S]-DPS synthesis conditions were as follows: SULT1A3 (10 μM), DP (3.0 mM), [^35^S]-PAPS (3.0 mM, SA = 4.3 μCi mmol^−1^), KPO_4_ (50 mM) pH = 7.4, 25 ºC, 16 h incubation. [^35^S]-DPS was purified using a PFPP HPLC column (see above). The UV spectrum of an [^35^S]-DPS sample was taken, and its concentration was determined from its specific activity using scintillation counting. The absorbance maximum of the spectrum at 284 nm corresponds to an Ɛ_284_ of 2.8 (±0.1) mM^-1^ cm^-1^.

### Dopamine metabolite detection in media

Transfectants are grown to 60 to 70% confluency at 37 °C in 12-well plates in the MEM containing neomycin (400 μg ml^−1^). The plates are washed (3×) with the PBS media (25 °C), and a fresh MEM containing neomycin and DP is added. After 24 h at 37 °C, 100 μl of media is removed and diluted 1:10 in water, and 250 μl of the dilute sample is loaded onto an Ultra PFPP column (150 × 4.6 mm length, 3-μm bead, Restek Corp.). DP metabolites (DP, DPS, HVA, DOPAC, and 3-MT) are separated using a 25-min, linear gradient from 100% buffer A (formic acid:water, 0.1% v/v) to 15% buffer B (formic acid:acetonitrile, 0.1% v/v). DP metabolite peaks are baseline separated and detected optically at 280 nm (see Fig. S1). Standard curves were used to quantitate each metabolite (Ɛ_280_ = 3.2 and 2.8 mM^-1^ cm^-1^ for DP and DPS, respectively). DPS was detected only in the media of cells that express SULT1A3. HVA, DOPAC, and 3-MT were not detectable (i.e., ≤1.0 μM) and total DP metabolite recovery was ≥95%.

## Data availability

All data and materials are available upon request (email: tom.leyh@einsteinmed.org).

## Conflict of interest

A provisional patent covering the compounds described in this manuscript has been submitted (US patent application number PCT/US2019/065442). All authors listed on the masthead are co-inventors on the patent.

## References

[1] Merikangas K.R., He J.P., Burstein M., Swanson S.A., Avenevoli S., Cui L., Benjet C., Georgiades K., Swendsen J. (2010). Lifetime prevalence of mental disorders in U.S. adolescents: results from the National Comorbidity Survey Replication--Adolescent Supplement (NCS-A). J. Am. Acad. Child. Adolesc. Psychiatry.

[2] Chesney E., Goodwin G.M., Fazel S. (2014). Risks of all-cause and suicide mortality in mental disorders: a meta-review. World Psychiatry.

[3] Mathers C.D., Loncar D. (2006). Projections of global mortality and burden of disease from 2002 to 2030. PLoS Med..

[4] Souery D., Amsterdam J., de Montigny C., Lecrubier Y., Montgomery S., Lipp O., Racagni G., Zohar J., Mendlewicz J. (1999). Treatment resistant depression: methodological overview and operational criteria. Eur. Neuropsychopharmacol..

[5] Thomas S.J., Shin M., McInnis M.G., Bostwick J.R. (2015). Combination therapy with monoamine oxidase inhibitors and other antidepressants or stimulants: strategies for the management of treatment-resistant depression. Pharmacotherapy.

[6] Olgiati P., Serretti A., Souery D., Dold M., Kasper S., Montgomery S., Zohar J., Mendlewicz J. (2018). Early improvement and response to antidepressant medications in adults with major depressive disorder. Meta-analysis and study of a sample with treatment-resistant depression. J. Affect Disord..

[7] Berton O., Nestler E.J. (2006). New approaches to antidepressant drug discovery: beyond monoamines. Nat. Rev. Neurosci..

[8] Suominen T., Uutela P., Ketola R.A., Bergquist J., Hillered L., Finel M., Zhang H., Laakso A., Kostiainen R. (2013). Determination of serotonin and dopamine metabolites in human brain microdialysis and cerebrospinal fluid samples by UPLC-MS/MS: discovery of intact glucuronide and sulfate conjugates. PLoS One.

[9] Lu J.H., Li H.T., Liu M.C., Zhang J.P., Li M., An X.M., Chang W.R. (2005). Crystal structure of human sulfotransferase SULT1A3 in complex with dopamine and 3'-phosphoadenosine 5'-phosphate. Biochem. Biophys. Res. Commun..

[10] Dajani R., Hood A.M., Coughtrie M.W. (1998). A single amino acid, glu146, governs the substrate specificity of a human dopamine sulfotransferase. SULT1A3. Mol. Pharmacol..

[11] Nowell S., Falany C.N. (2006). Pharmacogenetics of human cytosolic sulfotransferases. Oncogene.

[12] Falany J.L., Pilloff D.E., Leyh T.S., Falany C.N. (2006). Sulfation of raloxifene and 4-hydroxytamoxifen by human cytosolic sulfotransferases. Drug Metab. Dispos..

[13] Cook I.T., Duniac-Dmuchowski Z., Kocarek T.A., Runge-Morris M., Falany C.N. (2009). 24-Hydroxycholesterol sulfation by human cytosolic sulfotransferases: formation of monosulfates and disulfates, molecular modeling, sulfatase sensitivity and inhibition of LXR activation. Drug Metab. Dispos..

[14] Cook I., Wang T., Wang W., Kopp F., Wu P., Leyh T.S. (2016). Controlling sulfuryl-transfer biology. Cell Chem. Biol..

[15] Darrah K., Wang T., Cook I., Cacace M., Deiters A., Leyh T.S. (2019). Allosteres to regulate neurotransmitter sulfonation. J. Biol. Chem..

[16] Cook I., Wang T., Leyh T.S. (2019). Isoform-specific therapeutic control of sulfonation in humans. Biochem. Pharmacol..

[17] Cook I., Wang T., Leyh T.S. (2015). Sulfotransferase 1A1 substrate selectivity: a molecular clamp mechanism. Biochemistry.

[18] Cook I., Wang T., Almo S.C., Kim J., Falany C.N., Leyh T.S. (2013). Testing the sulfotransferase molecular pore hypothesis. J. Biol. Chem..

[19] Cook I., Wang T., Almo S.C., Kim J., Falany C.N., Leyh T.S. (2013). The gate that governs sulfotransferase selectivity. Biochemistry.

[20] Wang T., Cook I., Falany C.N., Leyh T.S. (2014). Paradigms of sulfotransferase catalysis: the mechanism of SULT2A1. J. Biol. Chem..

[21] Cook I., Wang T., Falany C.N., Leyh T.S. (2012). A nucleotide-gated molecular pore selects sulfotransferase substrates. Biochemistry.

[22] Wang T., Cook I., Leyh T.S. (2014). 3'-Phosphoadenosine 5'-phosphosulfate allosterically regulates sulfotransferase turnover. Biochemistry.

[23] Wang T., Cook I., Leyh T.S. (2016). Isozyme specific allosteric regulation of human sulfotransferase 1A1. Biochemistry.

[24] Eyring H. (1934). The activated complex in chemical reactions. J. Chem. Phys..

[25] Eberhardt E.S., Raines R.T. (1994). Amide-amide and amide-water hydrogen bonds: implications for protein folding and stability. J. Am. Chem. Soc..

[26] Case D.A., Babin V., Berryman J.T., Betz R.M., R. M., Cai Q., Cerutti D.S., Cheatham T.E., T.A. D., Duke R.E., Gohlke H., Goetz A.W., Gusarov S., Homeyer N., Janowski P. (2014). AMBER 14.

[27] Riches Z., Stanley E.L., Bloomer J.C., Coughtrie M.W. (2009). Quantitative evaluation of the expression and activity of five major sulfotransferases (SULTs) in human tissues: the SULT "pie. Drug Metab. Dispos..

[28] Wang T., Cook I., Leyh T.S. (2017). The NSAID allosteric site of human cytosolic sulfotransferases. J. Biol. Chem..

[29] Weisz J., Fritz-Wolz G., Gestl S., Clawson G.A., Creveling C.R., Liehr J.G., Dabbs D. (2000). Nuclear localization of catechol-O-methyltransferase in neoplastic and nonneoplastic mammary epithelial cells. Am. J. Pathol..

[30] Falany J.L., Falany C.N. (1996). Expression of cytosolic sulfotransferases in normal mammary epithelial cells and breast cancer cell lines. Cancer Res..

[31] Salman E.D., Kadlubar S.A., Falany C.N. (2009). Expression and localization of cytosolic sulfotransferase (SULT) 1A1 and SULT1A3 in normal human brain. Drug Metab. Dispos..

[32] Eisenhofer G., Coughtrie M.W., Goldstein D.S. (1999). Dopamine sulphate: an enigma resolved. Clin. Exp. Pharmacol. Physiol. Suppl..

[33] Stewart M.J., Watson I.D. (1983). Standard units for expressing drug concentrations in biological fluids. Br. J. Clin. Pharmacol..

[34] Zhu W., Xu H., Wang S.W., Hu M. (2010). Breast cancer resistance protein (BCRP) and sulfotransferases contribute significantly to the disposition of genistein in mouse intestine. AAPS J..

[35] Cook I.T., Duniec-Dmuchowski Z., Kocarek T.A., Runge-Morris M., Falany C.N. (2009). 24-hydroxycholesterol sulfation by human cytosolic sulfotransferases: formation of monosulfates and disulfates, molecular modeling, sulfatase sensitivity, and inhibition of liver x receptor activation. Drug Metab. Dispos..

[36] Sun M., Leyh T.S. (2010). The human estrogen sulfotransferase: a half-site reactive enzyme. Biochemistry.

[37] Baker J., Wolinski K., Malagoli M., Kinghorn D., Wolinski P., Magyarfalvi G., Saebo S., Janowski T., Pulay P. (2009). Quantum chemistry in parallel with PQS. J. Comput. Chem..

[38] Cook I., Wang T., Falany C.N., Leyh T.S. (2015). The allosteric binding sites of sulfotransferase 1A1. Drug Metab. Dispos..

[39] Arnold K., Bordoli L., Kopp J., Schwede T. (2006). The SWISS-MODEL workspace: a web-based environment for protein structure homology modelling. Bioinformatics.

[40] Berendsen H.J.C., Vanderspoel D., Vandrunen R. (1995). Gromacs - a message-passing parallel molecular-dynamics implementation. Comput. Phys. Commun..

[41] Nurisso A., Bravo J., Carrupt P.A., Daina A. (2012). Molecular docking using the molecular lipophilicity potential as hydrophobic descriptor: impact on GOLD docking performance. J. Chem. Inf. Model..

[42] Verdonk M.L., Cole J.C., Hartshorn M.J., Murray C.W., Taylor R.D. (2003). Improved protein-ligand docking using GOLD. Proteins.

[43] Whiteley C.G. (1999). Enzyme kinetics: partial and complete non-competitive inhibition. Biochem. Education.

[44] Grant G.A. (2018). The many faces of partial inhibition: revealing imposters with graphical analysis. Arch. Biochem. Biophys..

[45] Bradford M.M. (1976). A rapid and sensitive method for the quantitation of microgram quantities of protein utilizing the principle of protein-dye binding. Anal. Biochem..

