# Supplementary material for: Small-molecule control of neurotransmitter sulfonation
Source: J Biol Chem. 2020 Nov 24;296:100094. doi: 10.1074/jbc.RA120.015177 (PMC7948405; doi:10.1074/jbc.RA120.015177)
Supplement: Supplementary Information [file mmc1.pdf]

Controlling neurotransmitter sulfonation

Ian Cook[§], Mary Cacace[†], Ting Wang[§‡], Kristie Darrah[†], Alexander Deiters[°†] and Thomas S. Leyh[§*]

[†]*Department of Chemistry, University of Pittsburgh, PA 15260.* [§]*Department of Microbiology and Immunology, Albert Einstein College of Medicine, 1300 Morris Park Ave, Bronx, New York 10461-1926.*

*Supported by the National Institutes of Health Grants GM112728°, GM121849** and *GM127144**

[*]*Corresponding Author*

[‡]Current Address: *Drug Metabolism and Pharmacokinetics Department, Boehringer Ingelheim, Danbury, CT*

Key Words: catecholamine, neurotransmitter, sulfotransferase, allosteric, inhibitor, mechanism, synthesis, SULT1A3, isozyme, molecular dynamics, GROMACS, human mammary epithelial cells, structure activity relationship

Address: The Department of Microbiology and Immunology
Albert Einstein College of Medicine
1300 Morris Park Ave.
Bronx, New York 10461-1926
Phone: 718-430-2857
Fax: 718-430-8711
E-mail: tom.leyh@einsteinmed.org

Running Title: *Regulating neurotransmitter sulfonation in human cell lines*

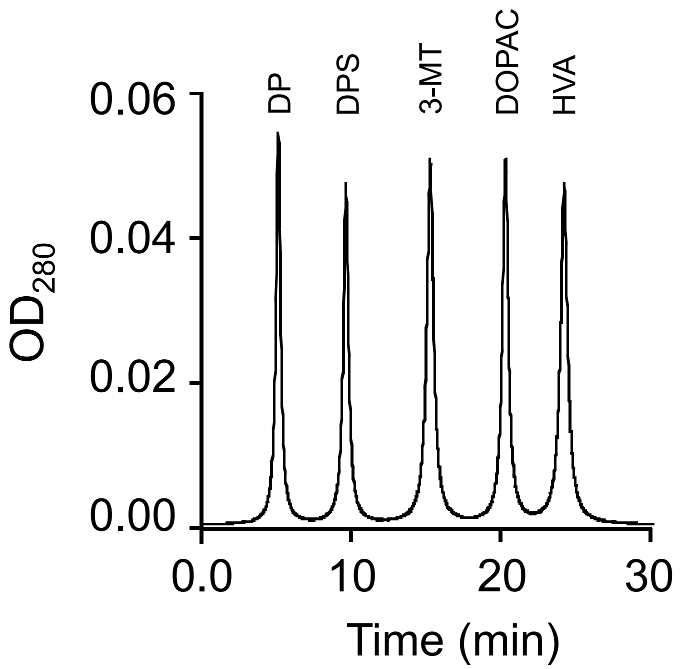

Figure S1. *HPLC separation of dopamine metabolites.* 250 µl of a solution containing 50 µM DP, DPS, DOPAC, 3-MT and HVA in water/formic acid (0.1% v/v) was loaded onto a PFPP column and separated as described in *Methods*. Metabolites were detected optically at 280 nM.

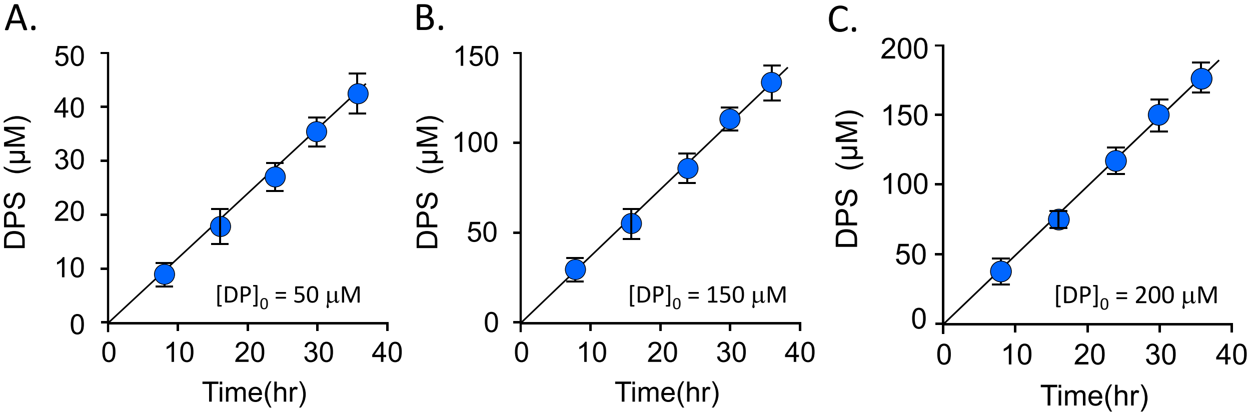

Figure S2. *Time dependence of DPS formation*. DP was added at the indicated concentrations (50, 150 and 200 µM) to the growth medium of 60-70% confluent HME-(+) cells, and the DPS concentration in the medium was determined at the indicated time intervals (see, *Materials and Methods*). DP and DPS constituted ≥ 95% of total added dopamine at all time points. Each data point in *Panels A - C* represents the average of three independent determinations.

Syntheses

Compound 12

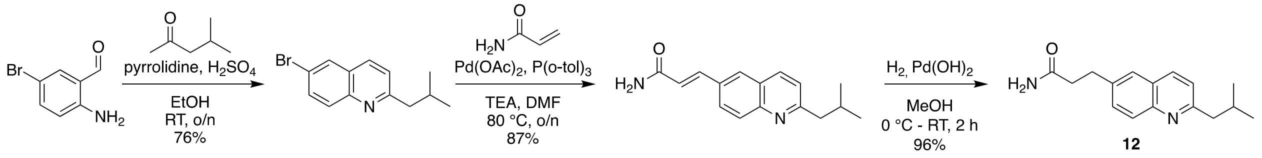

Compound **12** was synthesized from commercially available 2-amino-5-bromobenzaldehyde. A Friedlander reaction was used to generate the 6-bromo-2-isobutylquinoline intermediate**,** which was subsequently converted to the unsaturated amide through a palladium-catalyzed Heck coupling. Hydrogenation of the unsaturated amide afforded compound 12 in 96% yield.

Compound 13

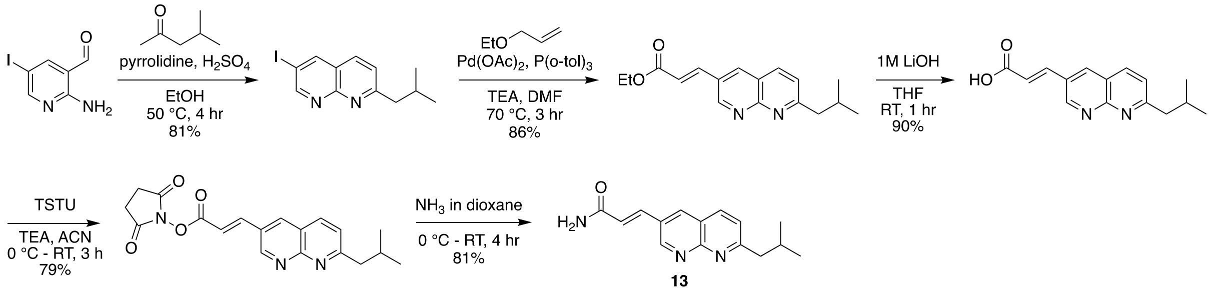

Compound **13** was synthesized from commercially available 2-amino-5-iodopyridine-3-carbaldehyde. First, a Friedlander reaction was used to generate the 6-iodo-2-isobutylnaphthalene. The unsaturated ester was obtained through a Heck coupling and subsequent ester hydrolysis generated the acrylic acid. The unsaturated amide intermediate was synthesized through the *N*-hydroxysuccinimide ester intermediate, which was subsequently hydrogenated to afford the final compound 13 in 45% yield.

Experimental Protocols

All reactions were performed in flame dried glassware and stirred magnetically. All starting materials were used from commercial sources without further purification. All reactions were monitored by thin layer chromatography (TLC), unless otherwise indicated. ^{1}H and ^{13}C NMR were performed on Bruker Ultrashield 400 MHz or 500 MHz instruments. High Resolution Mass Spectrometry was performed by the University of Pittsburgh facilities.

6-Bromo-2-isobutylquinoline. 2-Amino-5-bromobenzaldehyde (110 mg, 0.500 mmol) was dissolved in EtOH (800 μL) at room temperature. Pyrrolidine (45 μL, 0.550 mmol) and then concentrated sulfuric

acid (8 µL, 0.150 mmol) were added to the reaction mixture while stirring. 4-Methyl-2-pentanone (69 µL, 0.550 mmol) was added dropwise. After overnight stirring, the reaction was concentrated *in vacuo*. The yellow-brown residue was diluted with EtOAc and purified by flash chromatography on silica gel using 12% EtOAc in hexanes to yield a yellow solid (117 mg, 76%). ^{1}H-NMR (400 MHz, CDCl3) δ 7.96 (d, J= 8.8 Hz,1H), 7.93 (d, J =2.4 Hz,1H), 7.91 (d, J = 8.8 Hz,1H), 7.74 (dd, J = 8.8 Hz, 2 Hz, 1H), 7.28 (d,J = 8.4 Hz, 1H), 2.83 (d, J = 7.2 Hz, 2H), 2.20 (m, 1H), 0.97 (d, J = 6.8 Hz, 6H). 13C-NMR (500 MHz, CDCl3) δ 162.9, 146.7, 135.0, 132.8, 130.9, 129.6, 128.0, 123.0, 119.5, 48.4, 29.5, 22.7. HRMS calcd for $[M+H]^+$ 264.039, found 264.0398.s

(*E*)-3-(2-Isobutylquinolin-6-yl)acrylamide. 6-Bromo-2-isobutylquinoline (116 mg, 0.439 mmol) was dissolved in DMF (2 mL) at room temperature before palladium (II) acetate (9.8 mg, 0.044 mmol) and tri(*o*-tolyl)phosphine (54 mg, 0.176 mmol) were added sequentially. Triethylamine (620 µL, 4.39 mmol) and acrylamide (93 mg, 1.31 mmol) were added and the reaction stirred at 80 °C overnight. The reaction was cooled to room temperature and diluted with EtOAc (15 mL) and water (30 mL). The aqueous layer was extracted with EtOAc (3 x 15 mL). The organic layers were combined, washed with brine (30 mL), dried over anhydrous Na_2SO_4 (10 g), filtered, and concentrated *in vacuo*. The brown oil was re-dissolved in DCM and purified by flash chromatography on silica gel using 2.5% MeOH in DCM to yield an off-white solid (104 mg, 87%). ^{1}H NMR (400 MHz, $CDCl_3$) δ 8.07 (d, J = 8.4 Hz, 1H), 8.04 (d, J = 9.2 Hz, 1H), 7.88 (m, 2H), 7.84 (d, J = 15.6 Hz, 1H), 7.31 (d, J = 5.2 Hz, 1H), 6.59 (d, J = 15.6 Hz, 2H), 5.59 (br s, 2H), 2.86 (d, J = 7.2 Hz, 2H), 2.22 (m, 1H), 0.98 (d, J = 6.8 Hz, 6H). ^{13}C-NMR (400 MHz, $CDCl_3$) δ 167.4, 163.5, 142.1, 136.3, 131.8, 129.7, 129.0, 127.0, 126.7, 122.8, 119.9, 48.3, 29.5, 22.6. HRMS calcd for $[M+H]^+$ 254.14, found 255.14889.

3-(2-Isobutylquinolin-6-yl)propenamide (12). (*E*)-(2-Isobutylquinolin-6-yl)acrylamide (50 mg, 0.197 mmol) was dissolved in MeOH (8 mL) and the reaction mixture was cooled to 0 °C. Palladium hydroxide (6 mg, 0.039 mmol) was added to the reaction mixture while stirring. Hydrogen was bubbled through the reaction using a balloon as it stirred to room temperature over two hours. The reaction mixture was filtered over celite, concentrated *in vacuo*, and purified by flash chromatography on silica gel using 5% MeOH in DCM to afford a light-yellow solid (48 mg, 96%). ^{1}H-NMR (400 MHz, $CDCl_3$) δ 8.00 (d, J = 8.0 Hz, 1H), 7.99 (d, J = 8.4 Hz, 1H), 7.60 (br s, 1H), 7.56 (dd, J = 2.0 Hz, 1.6 Hz, 1H), 7.24 (s, 1H), 3.17 (t, J = 7.6 Hz, 2H) 2.84 (d, J = 7.2 Hz, 2H) 2.64 (t, J = 7.6 Hz, 2H), 2.19 (m, 1H), 0.97 (d, J = 6.4 Hz, 6H).^{13}C-NMR (400 MHz, $CDCl_3$) δ 173.9, 161.8, 146.8, 138.1, 135.6, 130.3, 129.0, 126.7, 126.2, 122.2, 48.2, 37.2, 31.2, 29.5, 22.5. HRMS calcd for $[M+H]^+$ 256.35, found 257.16512.

6-Iodo-2-isobutylnaphthyridine. 2-Amino-5-iodopyridine-3-carbaldehyde (80 mg, 0.323 mmol) was dissolved in EtOH (1.5 mL) at room temperature. Pyrrolidine (30 µL, 0.355 mmol) and concentrated sulfuric acid (5.2 µL, 0.097 mmol) were added sequentially. 4-Methyl-2-pentanone (44 µL, 0.355 mmol) was added last and the reaction stirred at 50 °C for five hours before it was concentrated *in vacuo*. The resulting brown solid was purified by flash chromatography on silica gel using 20% EtOAc in hexanes to yield a pale yellow solid (82 mg, 81%). ^{1}H-NMR (400 MHz, $CDCl_3$) δ 9.22 (d, J = 2 Hz, 1H), 8.54 (d, J = 2 Hz, 1H), 8.04 (d, J = 6.8 Hz, 1H), 7.41 (d, J = 6.8 Hz, 1H), 2.97 (d, J = 6 Hz, 2H), 2.32 (m, 1H),

0.99 (d, J = 5.6 Hz, 1H). ^{13}C-NMR (400 MHz, $CDCl_3$) δ 166.9, 158.6, 154.4, 150.9, 144.2, 135.5, 123.7, 122.8, 89.5, 48.2, 29.1, 22.5. HRMS calcd for $[M+H]^+$ 312.01, found 313.01927.

***(E)*-3-(7-Isobutylnaphthyridin-3-yl)acrylate.** 6-iodo-2-isobutylnaphthyridine (60 mg, 0.192 mmol) was diluted in DMF (1 mL) at room temperature before palladium (II) acetate (4 mg, 0.119 mmol) and tri(*o*-tolyl)phosphine (23 mg, 0.077 mmol) were added sequentially. Triethylamine (270 µL, 1.92 mmol) and ethyl acrylate (42 µL, 0.384 mmol) were added before the reaction stirred at 70 ℃ for three hours. The crude reaction mixture was diluted with EtOAc (10 mL) and water (25 mL). The aqueous layer was extracted with EtOAc (3 x 10 mL). The organic layers were combined, washed with brine (25 mL), dried over anhydrous Na_2SO_4 (8 g), filtered over cotton, and concentrated *in vacuo*. The crude oil was re-dissolved in EtOAc and purified by flash chromatography on silica gel using 30% EtOAc in hexanes to yield a beige solid (43 mg, 86%). ^{1}H-NMR (400 MHz, $CDCl_3$) δ 9.20 (d, J = 2.4 Hz, 1H), 8.16 (d, J = 2.4 Hz, 1H), 8.06 (d, J = 8.4 Hz, 1H), 7.79 (d, J = 16 Hz, 1H), 6.63 (d, J = 16 Hz, 1H), 4.27 (q, J = 7.2 Hz, 2H), 2.88 (d, J = 7.2 Hz, 2H), 2.29 (m, 1H), 1.32 (t, J = 7.2 Hz, 3H), 0.94 (d, J = 6.8 Hz). ^{13}C-NMR δ (500 MHz, CDCl3) 167.4, 166.3, 156.3, 152.2, 140.4, 136.9, 135.7, 127.8, 123.9, 120.8, 120.4, 60.8, 48.4, 29.2, 22.5, 14.3. HRMS calcd for $[M+H]^+$ 284.15, found 285.15990.

(*E*)-3-(7-Isobutylnaphthyridin-3-yl)acrylic acid. (*E*)-3-(7-Isobutylnaphthyridin-3-yl)acrylate (42 mg, 0.148 mmol) was dissolved in THF (1.2 mL) at room temperature before 1 M LiOH (1.2 mL) was added dropwise with a syringe. The reaction stirred vigorously for one hour and was the brought to pH of 5 with 1 M HCl before being diluted with EtOAc (10 mL) and water (30 mL) The aqueous layer was extracted with EtOAc (3 x 10 mL). The organic layers were combined, washed with brine, dried over anhydrous Na_2SO_4 (10 g), filtered over cotton and concentrated *in vacuo* to yield a white solid (34 mg, 77%). ^{1}H-NMR (500 MHz, MeOD) δ 9.35 (d, J = 1.5 Hz, 1H), 8.61 (d, J = 2 Hz, 1H), 8.38 (d, J = 8.5 Hz, 1H), 7.89 (d, J = 16 Hz, 1H), 7.58 (d, J = 8.5 Hz, 1H), 6.82 (d, J = 16.5 Hz, 1H), 2.92 (d, J = 7.5 Hz, 2H), 2.28 (m, 1H), 1.00 (d, J = 6.5 Hz). ^{13}C-NMR δ (500 MHz, MeOD) 168.6, 156.6, 153.6, 141.6, 139.4, 137.7, 129.9, 125.3, 122.7, 122.3, 47.9, 30.5, 22.8. HRMS calcd for $[M+H]^+$ 256.12, found 257.12857.

2,5-Dioxopyrrolidin-1-yl(*E*)-3-(7-isobutylnaphthyridin-3-yl)acrylate. (*E*)-3-(7-Isobutylnaphthyridin-3-yl)acrylic acid (24 mg, 0.093 mmol) was used crude and diluted in acetonitrile (1 mL). Triethylamine (39 µL, 0.279 mmol) was added to the suspension, dissolved the acid, and the reaction mixture was brought to 0 ℃. Once at temperature, *N*,*N*,*N*',*N*'-tetramethyl-*O*-(*N*-succinimidyl)uranium tetrafluoroborate (31 mg, 0.102 mmol) was added and the reaction stirred from 0 ℃ to room temperature over two hours. The crude reaction mixture was concentrated *in vacuo*, re-dissolved in DCM, and purified by flash chromatography on silica gel using 1% MeOH in DCM to afford a white solid (26 mg, 79%). ^{1}H-NMR (400 MHz, $CDCl_3$) δ 9.23 (d, J = 2 Hz, 1H), 8.25 (d, J = 2.4 Hz, 1H), 8.10 (d, J = 8.4 Hz, 1H), 8.04 (d, J = 16 Hz, 1H), 7.38 (d, J = 8.4 Hz, 1H), 6.80 (d, J =16 Hz, 1H), 2.90 (d, J = 7.2 Hz, 2H), 2.84 (s, 4H), 2.28 (m, 1H), 0.95 (d, J = 6.8 Hz. 6H). ^{13}C-NMR (500 MHz, $CDCl_3$) δ 169.2, 168.4,

161.6, 156.9, 152.2, 145.8, 137.3, 137.0, 126.9, 124.3, 120.5, 114.2, 48.6, 38.7, 29.3, 22.7. HRMS calcd for $[M+H]^+$ 353.14, found 354.14688.

(*E*)-3-(7-Isobutylnaphthyridin-3-yl)acrylamide (13). 2,5-Dioxopyrrolidin-1-yl (*E*)-3-(7-isobutylnaphthyridin-3-yl)acrylate (25 mg, 0.071 mmol) was added to reaction vessel and brought to 0 ℃ before ammonia solution (0.5 M in dioxane, 1.5 mL) was added dropwise while stirring. The reaction mixture stirred to room temperature over four hours before it was concentrated *in vacuo*, and purified by flash chromatography on silica gel using 5% MeOH in DCM to yield an off-white solid (15 mg, 81%). ^{1}H-NMR (400 MHz, DMSO) δ 9.22 (d, J = 2.4 Hz, 1H), 8.56 (d, J = 2.4 Hz), 8.38 (d, J = 8.4 Hz, 1H), 7.66 (br s, 1H), 7.63 (d, J = 16 Hz, 1H), 7.56 (d, J = 8.4 Hz), 7.24 (br s, 1H), 6.91 (d, J = 16 Hz), 2.85 (d, J = 7.6 Hz, 2H) 2.24 (m, 1H), 0.94 (d, J = 6.8 Hz, 6H). ^{13}C-NMR (500 MHz, MeOD) δ 169.9, 168.4, 156.5, 153.1, 139.3, 138.2, 137.6, 130.2, 125.3, 124.5, 122.3, 30.5, 26.2, 22.8. HRMS calcd for $[M+H]^+$ 255.14, found 256.14444.
